# Transcriptomic-Wide Discovery of Direct and Indirect HuR RNA Targets in Activated CD4^+^ T Cells

**DOI:** 10.1371/journal.pone.0129321

**Published:** 2015-07-10

**Authors:** Patsharaporn Techasintana, J. Wade Davis, Matthew M. Gubin, Joseph D. Magee, Ulus Atasoy

**Affiliations:** 1 Department of Surgery, University of Missouri, Columbia, Missouri, United States of America; 2 Department of Molecular Microbiology and Immunology, University of Missouri, Columbia, Missouri, United States of America; 3 Department of Biostatistics, University of Missouri, Columbia, Missouri, United States of America; 4 Department of Pathology and Immunology, Washington University School of Medicine, St. Louis, Missouri, United States of America; 5 Dalton Cardiovascular Research Center, University of Missouri, Columbia, Missouri, United States of America; University of Surrey, UNITED KINGDOM

## Abstract

Due to poor correlation between steady state mRNA levels and protein product, purely transcriptomic profiling methods may miss genes posttranscriptionally regulated by RNA binding proteins (RBPs) and microRNAs (miRNAs). RNA immunoprecipitation (RIP) methods developed to identify in vivo targets of RBPs have greatly elucidated those mRNAs which may be regulated via transcript stability and translation. The RBP HuR (ELAVL1) and family members are major stabilizers of mRNA. Many labs have identified HuR mRNA targets; however, many of these analyses have been performed in cell lines and oftentimes are not independent biological replicates. Little is known about how HuR target mRNAs behave in conditional knock-out models. In the present work, we performed HuR RIP-Seq and RNA-Seq to investigate HuR direct and indirect targets using a novel conditional knock-out model of HuR genetic ablation during CD4+ T activation and Th2 differentiation. Using independent biological replicates, we generated a high coverage RIP-Seq data set (>160 million reads) that was analyzed using bioinformatics methods specifically designed to find direct mRNA targets in RIP-Seq data. Simultaneously, another set of independent biological replicates were sequenced by RNA-Seq (>425 million reads) to identify indirect HuR targets. These direct and indirect targets were combined to determine canonical pathways in CD4+ T cell activation and differentiation for which HuR plays an important role. We show that HuR may regulate genes in multiple canonical pathways involved in T cell activation especially the CD28 family signaling pathway. These data provide insights into potential HuR-regulated genes during T cell activation and immune mechanisms.

## Introduction

Due to poor correlation between steady state mRNA levels and protein products, traditional genome profiling techniques may be limiting. Eukaryotic mRNAs are regulated posttranscriptionally by RNA-binding proteins (RBPs) and microRNAs (miRNAs) at the levels of transcript stability and translation. To better understand the complex milieu of posttranscriptional gene regulation, investigators have developed various methods to identify mechanisms of gene regulation. RNA-immunoprecipitation applied to gene chips (RIP-Chip) or, more recently, to RNA-Seq (next generation deep sequencing) are two such methods that have revealed networks of posttranscriptionally regulated genes. These methods can be used to explore both known and novel transcripts.

The *elav* family of RBPs and HuR (*elavl1*) in particular play important roles in stabilization and translational recruitment of labile mRNA transcripts to heavy polysomes in order to facilitate protein product expression. Since HuR is a stabilizer RBP, many labs, including our own, have taken advantage of this fact and performed HuR RIP-Chip (or Seq) and RNA-Seq to identify mRNAs which may be coordinately regulated by HuR in a variety of biological systems [1–3],[4]. However, the vast majority of these analyses are not truly independent biological samples and they were mostly performed in transformed cell lines. Not as much is known about HuR regulation of mRNA target genes in primary cells *in vivo*.

An active area of HuR investigation is in immune system regulation, especially during CD4^+^ T cell activation and differentiation. When our group first cloned murine HuR (HuA), we demonstrated that T cell activation results in 14-fold increases in the total cellular HuR levels [5]. Other groups have shown that following T cell activation, HuR rapidly translocates to the cytoplasm [6]. Thus, we and others have hypothesized that HuR may facilitate T cell activation as well as naïve CD4^+^ T cell differentiation programs.

Naive CD4^+^ T cells can differentiate into different subsets, including Th1, Th2, Th17, Tfh and Treg, with unique signature transcription factors and different patterns of cytokine gene expression. HuR was previously described as a regulator of transcription factors and cytokines promoting CD4^+^ Th2 differentiation such as GATA-3 and IL-4 [7, 8]. Th2 cytokines, such as IL-4, IL-5 and IL-13 play major roles in allergic airway inflammation. However, other HuR target transcripts essential for Th2 differentiation, besides those identified previously, may have been overlooked. In order to address this, we took advantage of HuR RIP-Chip techniques to determine direct HuR target transcripts in Th2 differentiating cells. Additionally, RNA-Seq was performed in HuR knock-out (KO) vs. wild type control CD4^+^ cells under Th2 polarization to identify target transcripts which were altered in the absence of HuR. To our knowledge, this is the first time these techniques have been used with independent biological replicates from primary murine CD4^+^ T cells undergoing Th2 differentiation.

Finally, we combined the data obtained from HuR RIP-Seq and RNA-Seq in order to determine HuR direct and indirect target transcripts under Th2 polarizing conditions. Our analysis (>400 million reads) reveals both known HuR regulated transcripts as well as new, putative HuR-associated targets under Th2 conditions which are crucial for Th2 differentiation and cytokine production. Such data when corroborated with translational expression can delineate previously overlooked genes which are required for T cell activation and inflammation in diseases such as asthma.

## Material and Methods

### Mice

OX40 Cre HuR^fl/fl^ mice were generated as previously described [7]. In HuR^fl/fl^ mice, a portion of the promoter region, exon 1 and exon 2 of HuR gene were flanked with the loxP sites which allow HuR ablation to occur upon *cre* recombinase excision. These mice were then crossed to OX40-*cre* mice in which *cre* recombinase enzyme is expressed under the control of OX40 promoter in activated T cells to generate OX40-*cre* HuR^fl/fl^ KO mice. All mice used were on the C57BL/6 background and housed in the pathogen free facilities. All animal experiments and procedures were conducted in accordance with the guidelines set forth by the University of Missouri Animal Care and Use Committee (IACUC). The University of Missouri IACUC approved all the experiments in this manuscript.

### Murine T cell polarization *in vitro*

Naive splenocytes were isolated from 6-8-wk-old C57BL/6 OX40-*Cre* HuR^fl/fl^ knockout mice or C57BL/6 HuR^fl/fl^ control mice. CD4^+^ T cells were isolated using CD4 (L3T4) MicroBeads (Miltenyi Biotec) following the manufacturer’s protocol. Cells were activated with anti-CD3 anti-CD28 (5 μg/ml each) for 5 days in T cell media (DMEM, 10% FCS, 50ug/ml gentamicin, 1mM Na Pyruvate, 2mM L-Glutamine and 0.05mM beta-mercaptoethanol (2-BME). For Th2 polarizing condition 100 U/ml rIL-4, 50 U/ml rIL-2, and 10 μg/ml anti–IFN-γ Ab were added in the media upon activation. Anti-CD3, anti-CD28, anti-IL-4, and anti- IFN-γ antibodies were purchased from eBioscience (San Diego, CA). Cytokines were all purchased from Peprotech (Rocky Hill, NJ). Tissue culture reagents were all purchased from Life Technologies.

### RNA isolation and sequencing

CD4^+^ Th2 polarized cells were harvested from the *in vitro* culture on day 5 post-activation. Approximately, 1x10^7^ cells were lysed and re-suspended in 1 ml of Trizol reagent (Life Technologies). Total RNA was then isolated according to the company protocol. For RNA-sequencing, 5ug of RNA from seven OX40-*cre* HuR^fl/fl^ mice and seven HuR^fl/fl^ controls were used to make the libraries (TruSeq). RNA-sequencing was performed using an Illumina HiSeq 2000 sequencer at the DNA Core facility of the University of Missouri. These libraries were sequenced in two different runs (one 6-plex and one 8-plex) of one lane each using 100 bp single-end reads.

### RNA-immunoprecipitation (RIP)

HuR and IgG1 RNA-immunoprecipitations were performed according to published protocol [1, 9]. Briefly, CD4^+^ Th2 polarized cells from HuR^fl/fl^ wild-type controls on day 5 of *in vitro* polarization were lysed in polysomal lysis buffer (PLB) supplemented with RNaseOUT, DTT and complete protease inhibitor. HuR (clone 3A2) or IgG1 isotype control antibodies were pre-coated onto protein A Sapharose beads (PAS) overnight at 4°C. On the next day, antibody coated beads were washed with NT-2 buffer (50mM Tris-Hcl pH7.4, 150mM NaCl_2_, 1mM MgCl_2_ and 0.05%NP-40) at least 5 times. Equal amount of protein lysates in PLB buffer were then incubated with HuR or IgG1 antibody coated beads for 4 hours at 4°C. Beads were then washed with NT-2 buffer 7 times. After the last wash, the beads were then incubated with 20 units of RNase-free DNase I (15 min, 30°C) and washed once with NT-2 buffer. The ribonucleoprotein complexes were dissociated from the beads by incubating in 100 μl NT-2 buffer containing 0.1% SDS and 0.5 mg/ml Proteinase K (30 min, 55°C). The beads were then spun down and supernatants were collected. RNA extraction from the supernatant was performed with acid-phenol and precipitated using absolute ethanol. Total RNA was then processed for RNA-sequencing (RNA-Seq). From each biological replicate (n = 3), two libraries (HuR IP and IgG1 control) were prepared and sequenced on an Illumina HiSeq 2000 with 100 bp single end reads. Each library was sequenced using a full lane on the sequencer.

### Statistical analyses and bioinformatics

RNA-Seq data and RIP-Seq data were analyzed separately to determine the indirect and direct HuR targets, respectively. After identifying those targets, the lists were merged and used for further analysis to identify enriched canonical pathways and to build novel networks related to HuR in CD4^+^ T cells.

### RNA-Sequencing

The primary analysis of the pre-processed RNA-Seq data was conducted using R/Bioconductor, with other free, open-source programs used for the pre-processing itself. The preprocessing steps consisted of: 1) quality assessment of FASTQ files using FastQC (http://www.bioinformatics.bbsrc.ac.uk/projects/fastqc), 2) trimming of adapters from each library using Cutadapt, 3) aligning libraries to the mm10 genome (http://hgdownload-test.cse.ucsc.edu/goldenPath/mm10/bigZips/) using a seed-and-vote method, and 4) forming a read-count matrix at the gene level by read summarization via featureCounts in Rsubread.

Non-specific filtering of genes prior to statistical testing was carried out to increase the power of detecting differentially expressed genes [10], based on the requirement that a gene have an expression level greater than 1 count per million reads mapped (CPM) for at least 7 libraries across all samples (HuR KO and wild-type control). Library normalization to adjust for differences in library size was then made using the TMM method [11] in edgeR [12]. A flexible mean-variance modeling and transformation process known as voom [13], was used in conjunction with a linear modeling approach [14], a combination known as Limma Voom, which was recently shown to perform among the best of available techniques in an comprehensive comparison of differential gene expression methods for RNA-Seq data [15]. Using this framework, normalized expression levels were compared between the KO/CTL, resulting in estimated fold changes and associated p-values. Adjustment to the p-values was made to account for multiple testing using the false discovery rate (FDR) method of Benjamini and Hochberg; these are reported as q-values.

### RIP-Sequencing

Pre-processing and read summarization of RIP-Seq data was carried out as described for the RNA-Seq data. For each animal (n = 3), a HuR and IgG1 sample was processed separately using the RIPSeeker [16] algorithm, which is a *de novo* method for detecting peaks (indicating protein–RNA interactions) based on hidden Markov models (HMM). This is carried out in a multistage process whereby RIPSeeker first partitions the genome into non-overlapping bins, whose sizes are optimally determined per chromosome (optimal bin size selected was between 200–300 bp for our data), and each bin counts any reads that fall into it. Clearly, multiple (and often adjacent) bins may together correspond to a single RNA transcript that binds to HuR, so that the bins must be treated as dependent observations. This is dealt with via the HMM approach, which also takes advantage of the background signal information provided by the IgG1 sample, and is used to make a prediction of the state of the bin (RIP or background). To assess the significance of the prediction for a bin (i.e., that specific genomic region), both the RIP library and control library are assigned a RIPScore at each bin location. Adjacent regions with the same prediction are merged and their RIPscores are averaged across bins. Thus, by application of the central limit theorem, the difference between the average RIPscores (RIP and control) has been shown to have a Gaussian distribution, which can be used to derive a p-value. Finally, those p-values are adjusted for multiple testing using FDR. Data (HuR and IgG1 control) from each animal were analyzed individually by RIPSeeker to produce a list of annotated RIP regions (aka peaks) and associated q-values. Taking cues from the quality control protocol used for ChIP-Seq data in the ENCODE project, the peak lists were compared between animals, and excluded if the peak list of any sample overlapped by less than 50% with the other samples.

### Combined analysis of RNA-Seq and RIP-Seq results for canonical pathways and novel networks

The set of genes identified by RNA-Seq analysis (indirect targets) and by RIP-Seq analysis (direct targets) were merged and used for further analysis to identify enriched canonical pathways and to build novel networks related to HuR in CD4^+^ T cells. Canonical pathways were assessed and novel networks were generated through the use of Ingenuity Pathways Analysis (Ingenuity Systems, www.ingenuity.com), henceforth IPA, as previously described [17]. The complete lists of identified genes were uploaded into IPA along with the Entrez Gene identifier (ID), fold change, and q-value. In the event of duplicate genes prior to uploading into IPA, RIP-Seq (direct target) information for the gene was used as opposed to RNA-Seq (indirect target). Within IPA, in the event of commonly mapped IDs to the Ingenuity Knowledge Base (IKB), the maximum fold change (i.e., RIP-Seq values took precedence over RNA-Seq values when both significant) was used to represent the single results for that Gene ID. For canonical pathway analysis, Fisher’s exact test was used to determine which pathways are enriched for our reported direct or indirect HuR targets. For novel networks, a detailed description of the network generating algorithm is provided by Calvano et al. [18]. Graphical representations of the pathways were generated using IPA’s Path Designer, which illustrates the relationships between molecules (i.e., nodes), and the biological relationship between two nodes is represented as an edge (i.e., connecting line). All edges are supported by at least 1 reference stored in the IKB which was derived either from the literature, from a textbook or from canonical information.

### RT-qPCR

RNA isolation from CD4^+^ Th2 polarized cells on day 5 post-activation was performed using Trizol extraction following the manufacture’s protocol. Reverse transcription (RT) was performed using 0.5–1 ug of RNA with Superscript III reverse transcriptase (Invitrogen). Quantitative PCR was done in triplicate using Platinum SYBR green Universal (Invitrogen). The data were analyzed using comparative CT method with Gapdh as an endogenous control. Data were present in arbitrary relative quantitative ratio (RQ). Student’s t-test was used to calculate p-value based on delta-delta CT of the targets.

Murine primers for qPCR were listed below (forward/reverse):

Gapdh: 5′-TCAACAGCAACTCCCACTCT TCCA-3′/ 5′ACCCTGTTGCTGTAGCCGTATTCA-3′

*Il2*: 5′-CCCAAGCAGGCCACAGAATTGAAA-3′/

5′-AGTCAAATCCAGAACATGCCG CAG-3′

Camk2: 5′-TTGGCAGCAGACCCTAATG-3′/

5′-CTCCAAGCTGAGTGGACAAA-3′

Camk4: 5′-TCTCACACCCGAACATCATAAA-3′/

5′-CTGTAGTATCCCTTCTCCACAATC-3′

## Results

**Fig 1:**
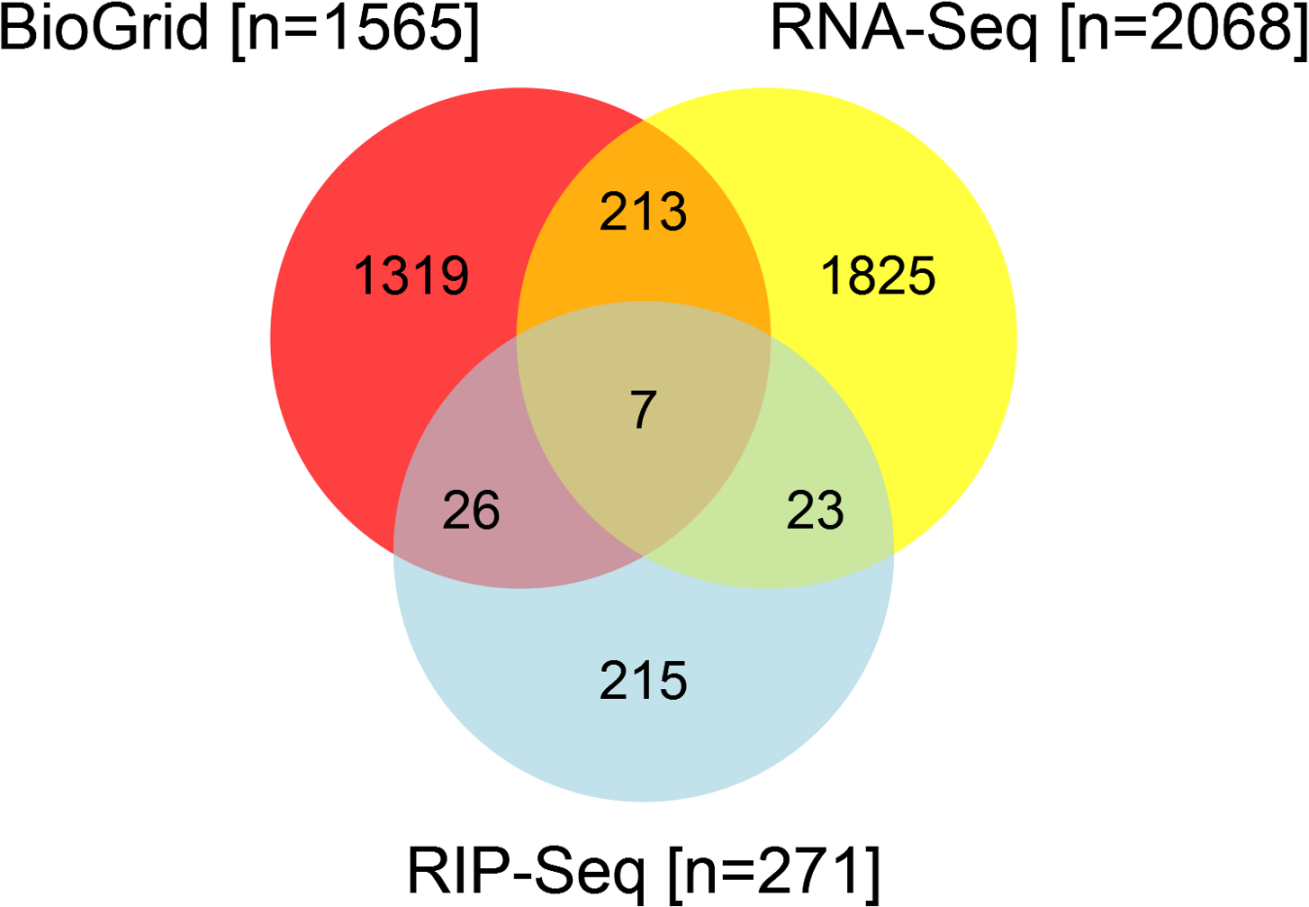
Venn diagram of RNA-Seq, RIP-Seq, and BioGRID reported ELAVL1 target genes. Results from the RNA-Seq and RIP-Seq experiments were compared to genes experimentally verified in the literature to interact with ELAVL1, as curated in the BioGRID database. Results from BioGRID were limited to genes with mouse orthologs to make the results more compatible with our experiments.

Our RNA-Seq experiments obtained approximately 425 million total raw reads (mean of 30 million), while the RIP-Seq experiments resulted in approximately 160 million total raw reads (mean of 26 million). Fig 1 provides a broad overview of the results found by RNA-Seq and RIP-Seq, in terms of the number of genes indicated as being significant by each method and how they are related. We searched BioGRID, a public database (http://thebiogrid.org/, accessed 6/24/2014) that aggregates and archives published genetic and protein interaction data from model organisms and humans [19–21], in order to compare our identified direct and indirect HuR (ELAVL1) targets with those experimentally determined in the literature. We downloaded data files containing approximately 2000 interactions involving HuR in human, mouse, and rat. Nearly all data came from humans; therefore, we only used BioGRID genes with mouse orthologs to construct Fig 1. We also limited our RNA-Seq and RIP-Seq findings to regions with official gene symbols because gene symbol is the identifier level at which the BioGRID data is reported.

BioGrid, RNA-Seq and HuR RIP-Seq revealed 1514, 2068 and 271 transcripts involving in Th2 differentiation respectively. Under Th2 polarizing conditions, 271 transcripts were shown to be direct HuR target transcripts by HuR RIP-seq. The RNA expression level of 30 genes shown to be direct HuR targets were altered in the absence of HuR. Of all the transcripts analyzed by these 3 methods, only seven have previously been shown to be known interacting targets by BioGrid. Thus, using these 3 analyses in combination, we discovered not only the potential target transcripts with which HuR may physically interact, but we also revealed the transcripts that may require HuR for their expression.

### HuR indirect targets in CD4^+^ Th2 differentiation identified by RNA-Seq

2080 genes (2068 with designated gene symbol in NCBI Gene) were found based on a nominal p-value cutoff of .05 (with corresponding q < .30), with a median p-value of .1275 (q = .15). The full listing of these genes is given in S1 Table. A broad entry criterion was selected because the ultimate goal was to use the identified differentially expressed genes as input for a combined network analysis, using both RNA-Seq and RIP-Seq results. The top 50 differentially expressed genes (with an assigned gene symbol) discovered via RNA-Seq are given in Table 1, sorted by the absolute magnitude of the fold change. Here, fold change is defined as knock-out (KO) divided by wild-type control (CTL).

**Table 1:** Top 50 RNA-Seq significantly different genes sorted by magnitude of fold change (KO/CTL). The official symbol of the top 50 (significant) genes found via RNA-Seq are given, along with fold change (KO/CTL), raw p-value for differential expression, and q (false discovery rate value based on method of Benjamini-Hochberg).

**Gene ID**	Symbol	Name	p	q	Fold
100862004	Erdr1	erythroid differentiation regulator 1	<0.01	<0.01	17.96
57738	Slc15a2	solute carrier family 15 (H+/peptide transporter), member 2	0.03	0.24	8.07
72469	Plcd3	phospholipase C, delta 3	<0.01	<0.01	0.15
338521	Fa2h	fatty acid 2-hydroxylase	0.01	0.14	3.29
246228	Vwa1	von Willebrand factor A domain containing 1	<0.01	<0.01	3.17
622402	Akr1c12	aldo-keto reductase family 1, member C12	<0.01	0.04	2.92
246779	Il27	interleukin 27	0.04	0.26	2.83
226654	Tstd1	thiosulfate sulfurtransferase (rhodanese)-like domain containing 1	<0.01	<0.01	2.78
74761	Mxra8	matrix-remodelling associated 8	<0.01	<0.01	2.77
105349	Akr1c18	aldo-keto reductase family 1, member C18	0.02	0.19	2.63
16590	Kit	kit oncogene	<0.01	<0.01	0.38
67405	Nts	neurotensin	0.02	0.19	2.59
16187	Il3	interleukin 3	0.02	0.17	2.55
100503024	Gm19512	predicted gene, 19512	<0.01	<0.01	2.53
78809	4930562C15Rik	RIKEN cDNA 4930562C15 gene	<0.01	0.01	2.52
100504267	Trp53cor1	tumor protein p53 pathway corepressor 1	<0.01	<0.01	0.41
16153	Il10	interleukin 10	0.01	0.13	2.40
20259	Scin	scinderin	<0.01	0.02	2.29
93695	Gpnmb	glycoprotein (transmembrane) nmb	<0.01	0.07	2.29
207375	Fam120c	family with sequence similarity 120, member C	<0.01	<0.01	2.29
77772	Dcst1	DC-STAMP domain containing 1	<0.01	0.06	2.27
13497	Drp2	dystrophin related protein 2	<0.01	<0.01	0.44
14419	Gal	galanin	0.03	0.24	2.26
106504	Stk38	serine/threonine kinase 38	<0.01	<0.01	2.23
16909	Lmo2	LIM domain only 2	0.01	0.10	2.16
20344	Selp	selectin, platelet	0.01	0.11	2.14
78309	Cul9	cullin 9	<0.01	<0.01	0.47
26549	Itgb1bp2	integrin beta 1 binding protein 2	<0.01	0.02	0.47
12484	Cd24a	CD24a antigen	0.03	0.23	2.13
19224	Ptgs1	prostaglandin-endoperoxide synthase 1	0.01	0.09	2.11
17067	Ly6c1	lymphocyte antigen 6 complex, locus C1	0.03	0.24	2.10
14586	Gfra2	glial cell line derived neurotrophic factor family receptor alpha 2	<0.01	0.05	2.09
15930	Ido1	indoleamine 2,3-dioxygenase 1	0.01	0.12	2.06
98256	Kmo	kynurenine 3-monooxygenase (kynurenine 3-hydroxylase)	<0.01	0.08	2.05
329002	Zfp236	zinc finger protein 236	<0.01	<0.01	0.49
71213	Cage1	cancer antigen 1	<0.01	<0.01	0.50
83382	Siglece	sialic acid binding Ig-like lectin E	0.01	0.11	0.50
12323	Camk2b	calcium/calmodulin-dependent protein kinase II, beta	<0.01	<0.01	0.50
383075	Enthd1	ENTH domain containing 1	<0.01	<0.01	0.50
20375	Sfpi1	SFFV proviral integration 1	0.03	0.22	1.97
74055	Plce1	phospholipase C, epsilon 1	<0.01	0.04	0.51
64011	Nrgn	Neurogranin	0.01	0.10	1.96
16177	Il1r1	interleukin 1 receptor, type I	0.02	0.19	1.96
66457	2810002D19Rik	RIKEN cDNA 2810002D19 gene	<0.01	<0.01	0.51
75828	Hormad2	HORMA domain containing 2	<0.01	0.06	1.95
16183	Il2	interleukin 2	0.01	0.10	1.93
239102	Zfhx2	zinc finger homeobox 2	<0.01	0.04	0.52
69479	1700029J07Rik	RIKEN cDNA 1700029J07 gene	<0.01	<0.01	0.52
210356	Nckap5	NCK-associated protein 5	0.03	0.22	1.91
18131	Notch3	notch 3	<0.01	0.01	0.52

Most of the genes in Table 1 are highly significant (median q = .045) with a large fold-change (median magnitude of the fold change = 2.15). We also determined efficiency of HuR ablation in activated HuR KO CD4^+^ T cells by Western analysis. As shown in S1D Fig, HuR levels in HuR KO CD4^+^ T cells are indeed abolished. Additionally, we also verified RNA-Seq data by determining mRNA steady state levels of three potential HuR target genes (CAMK2, CAMK4 and IL-2) by RT-qPCR. Consistent with the RNA-Seq data, IL-2 mRNA was increased in KO T cells by two-fold, whereas CAMK2 and CAMK4 levels were decreased 50% and 15%, respectively RT-qPCR (S1A–S1C Fig).

Many genes that are involved in CD4^+^ T cell activation and Th2 differentiation were identified as genes whose expression is altered in the absence of HuR as shown in Table 1. Only a few of those transcripts have been previously verified as HuR targets in other transformed cell lines such as Plcd3 and Trp53cor1 [22, 23].

Many of those known HuR regulated transcripts are not common with transcripts found in CD4^+^ T cells, even if those transcripts are highly expressed in both transformed cell lines and CD4^+^ T cells. This may indicate that HuR function may be cell type specific and HuR targets in transformed cell lines may be different from primary cells. The RNA-Seq data also showed novel HuR target transcripts encoding for cytokines, cytokine receptors and transcription factors required for CD4^+^ T cell functions such as IL-2, IL-10 and IL-1R1.

### Direct *in vivo* HuR targets identified by RIP-Seq in CD4^+^ Th2 differentiation

Three independent experiments were conducted and analyzed separately to determine the number of significant binding events (hits or peaks). Those regions were annotated based on which gene the region overlapped (or the gene with nearest start site). Experiment 1 identified 149 binding events that mapped to 81 genes, experiment 2 found 381 that mapped to 193 genes, and experiment 3 found 348 that mapped to 172 genes. Experiment 1 did not meet the minimum agreement with the other two experiments, so those results were excluded from further analysis. The regions from the remaining two experiments were aggregated at the gene level for a total of 271 unique genes with at least one significant binding event overlapping them (S2 Table). The top 100 significantly different annotated regions from among 271 identified via RIP-Seq are given in Table 2, along with the total number of significant ‘hits’ (binding events) at that region among two independent RIP experiments.

**Table 2:** Top 100 significant RIP-Seq regions arranged by gene type. The top 100 significantly different annotated regions from among 271 identified via RIP-Seq are given along with the total number of significant ‘hits’ (binding events) at that region among two independent RIP experiments. Fold denotes the *minimum* fold change observed across all of the hits, where fold change is expressed as (HuR/IgG). All regions given in this table have a *minimum* q-value (false discovery rate value based on method of Benjamini-Hochberg) <1E-99 from testing for HuR binding to RNA. The regions are arranged by gene type, and then sorted by fold change. Location of binding overlaps the annotated region unless otherwise noted.

Symbol	Feature Type	Hits	Fold ≥	Symbol	Feature Type	Hits	Fold ≥
Adipor2*	protein coding	2	714	Odf2	protein coding	5	149
Nfe2l3*	protein coding	2	510	Tcp11l1*	protein coding	2	146
Ttc7b*	protein coding	5	500	Gm1043*	protein coding	1	145
Atxn1	protein coding	1	496	Mrgprd*	protein coding	5	138
Mapk14*	protein coding	3	477	Fgf23*	protein coding	3	137
Cdk6	protein coding	1	473	Fam133b*	protein coding	1	133
Olfr1445*	protein coding	3	464	Cpne6*	protein coding	6	116
Clrn1*	protein coding	2	443	Wfdc8	protein coding	3	92
9130011E15Rik	protein coding	3	397	Hrasls5*	protein coding	3	86
Abcc2*	protein coding	2	390	Gm15155	protein coding	4	84
Wnt16*	protein coding	3	368	Adam29*	protein coding	6	82
Nrn1*	protein coding	2	363	Mex3b*	protein coding	3	80
Atp2b4*	protein coding	3	357	Kctd21*	protein coding	2	78
Kcnh5*	protein coding	2	352	Prr3	protein coding	3	76
Cldn25*	protein coding	1	346	Tfdp1	protein coding	4	70
9630033F20Rik	protein coding	1	316	Gm5072*	protein coding	3	66
Glb1*	protein coding	2	314	Fam107b	protein coding	5	62
Exoc2	protein coding	2	309	Hck*	protein coding	2	57
Adam12	protein coding	1	300	Tmx1*	protein coding	2	52
Fam199x	protein coding	2	285	Cd47	protein coding	5	51
Slc8a3	protein coding	3	281	Disp1*	protein coding	3	42
Nup210l	protein coding	3	276	Zc3h7a	protein coding	2	31
Adh7*	protein coding	1	273	Dtnbp1*	protein coding	2	25
Ccin*	protein coding	2	269	Ctdspl2	protein coding	2	20
Foxr1*	protein coding	1	267	Gm11383*	pseudogene	1	1377
Kcnb1	protein coding	5	267	RP23-331E4.1*	pseudogene	4	281
Zfhx3*	protein coding	2	251	Gm13567*	pseudogene	1	234
Mettl13*	protein coding	2	244	Gm13260*	pseudogene	1	113
Dock9*	protein coding	1	242	Gm13400*	pseudogene	3	77
Tcp11l2*	protein coding	3	238	Rps2-ps6*	pseudogene	4	13
Mbnl1*	protein coding	6	235	Gm14152	lincRNA gene	2	353
Kcnt2*	protein coding	3	213	Gm12596*	lincRNA gene	3	316
Espl1	protein coding	6	202	Gm13391*	lincRNA gene	3	296
Dag1*	protein coding	2	193	E530001K10Rik	lincRNA gene	1	205
Dram2	protein coding	3	191	AC169129.1	lincRNA gene	3	48
Ndufa4l2*	protein coding	3	191	Gm26333*	miRNA gene	2	361
Pola1	protein coding	4	187	Gm25947*	miRNA gene	3	253
Camk1*	protein coding	1	185	Mir1956*	miRNA gene	2	109
Srxn1*	protein coding	3	171	Gm25972*	miRNA gene	3	85
Tbl1xr1*	protein coding	4	170	Gm24179*	snoRNA gene	4	350
Fam174a*	protein coding	4	169	Gm23404*	snoRNA gene	8	125
Ebf2	protein coding	4	168	Gm24539*	snoRNA gene	3	27
Elavl1	protein coding	6	159	Gm22983*	snRNA gene	2	403
Sycp1	protein coding	7	158	Gm25130*	snRNA gene	2	78
Gm8914*	protein coding	2	157	Gm22628*	snRNA gene	2	22
Tram1*	protein coding	6	157	4930505M18Rik*	unclassified gene	1	1652
Shoc2	protein coding	8	155	AA387200*	unclassified gene	2	142
Cdh13*	protein coding	4	154	Gm14720	unclassified gene	3	106
Pold3*	protein coding	2	153	Gm13256*	unclassified gene	5	48
Golga1*	protein coding	2	151	C330013F16Rik	unclassified ncRNA	2	361

*Denotes the nearest annotated gene to the discovered binding site, rather than an overlapping annotated gene.

The regions are arranged by gene type, and then sorted by fold change. Because each hit has an associated p-value, q-value, and fold change (HuR/IgG), we conservatively reported the minimum fold change within a gene, so that all hits within a gene have a fold change as least as large as the one reported. Similarly, we conservatively report the maximum p-value and q-value, so that all hits within a gene have a value at least as small as the one reported. All regions given in Table 2 have a minimum q-value <1E-99 from testing for HuR binding to RNA, so we have suppressed reporting the p-value and q-value for Table 2, but the values are given in S2 Table. Location of binding overlaps the annotated region unless otherwise noted, in which case the reported gene is the nearest annotated gene; we have indicated those in Table 2 and S2 Table.

Various transcripts of protein coding genes, pseudogenes, lincRNA genes, miRNA genes, snoRNA genes and unclassified genes were found to physically interact with HuR by HuR RIP-Seq. The top significant HuR associated transcripts and the number of HuR hits are displayed in Table 2. Many of those transcripts are novel HuR target genes which have been shown to play pivotal roles in CD4^+^ T cell activation and Th2 differentiation. We validated HuR RIP-Seq data by performing HuR RIP followed by RT-qPCR in some of potential HuR transcripts in Th2 polarized CD4^+^ T cells. IL-2, CAMK2 and CAMK4 transcript enrichment in RIP using HuR antibody were analyzed as compared to RIP using IgG1 antibody. In agreement with the HuR RIP-Seq data, IL-2 and CAMK4 were found to be direct HuR target transcripts while CAMK2 did not seem to be an appreciable direct HuR target transcript (S2A Fig).

### Combined network analysis of RNA-Seq and RIP-Seq regions

Table 3 lists alphabetically the seven genes found in the intersection of Fig 1, and combines information reported in Tables 1 and 2 and S2 Table. In two instances, the hits do not perfectly overlap the annotated gene, so the reported gene is the nearest annotated gene; we have indicated those in Table 3.

**Table 3:** Genes found in both RNA-Seq and RIP-Seq, sorted alphabetically. The seven protein-coding genes identified as significant based on RIP-Seq and RNA-Seq are given. For RIP-Seq, the total number of significant ‘hits’ (binding events) per gene are reported, counted across two independent RIP experiments. Fold denotes the *minimum* fold change observed across all of the hits, where fold change is expressed as (HuR/IgG), and q denotes the *maximum* q-value (false discovery rate value based on method of Benjamini-Hochberg) from testing for HuR binding to RNA. Location of binding overlaps the annotated gene unless otherwise noted. For RNA-Seq, fold denotes change (HuR KO/CTL).

			RIP-Seq			RNA-Seq		
Gene ID	Symbol	Name	Fold ≥	q	Hits	AveExpr	q	Fold
12326	Camk4	calcium/calmodulin-dependent protein kinase IV	637	<1E-68	2	7.40	.060	0.845
16423	Cd47	CD47 antigen (Rh-related antigen, integrin-associated signal transducer)	51	<1E-99	5	8.71	.077	1.181
15568	Elavl1	ELAV (embryonic lethal, abnormal vision)-like 1 (Hu antigen R)	159	<1E-99	6	7.25	.002	1.316
67698	Fam174a*	family with sequence similarity 174, member A	169	<1E-99	4	4.70	.111	1.111
18286	Odf2	outer dense fiber of sperm tails 2	149	<1E-99	5	7.46	.041	0.864
320554	Tcp11l1*	t-complex 11 like 1	146	<1E-99	2	0.49	.073	1.632
106205	Zc3h7a	zinc finger CCCH type containing 7 A	31	<1E-99	2	7.59	.002	1.308

*Denotes the nearest annotated gene to the discovered binding site, rather than an overlapping annotated gene.

**Fig 2:**
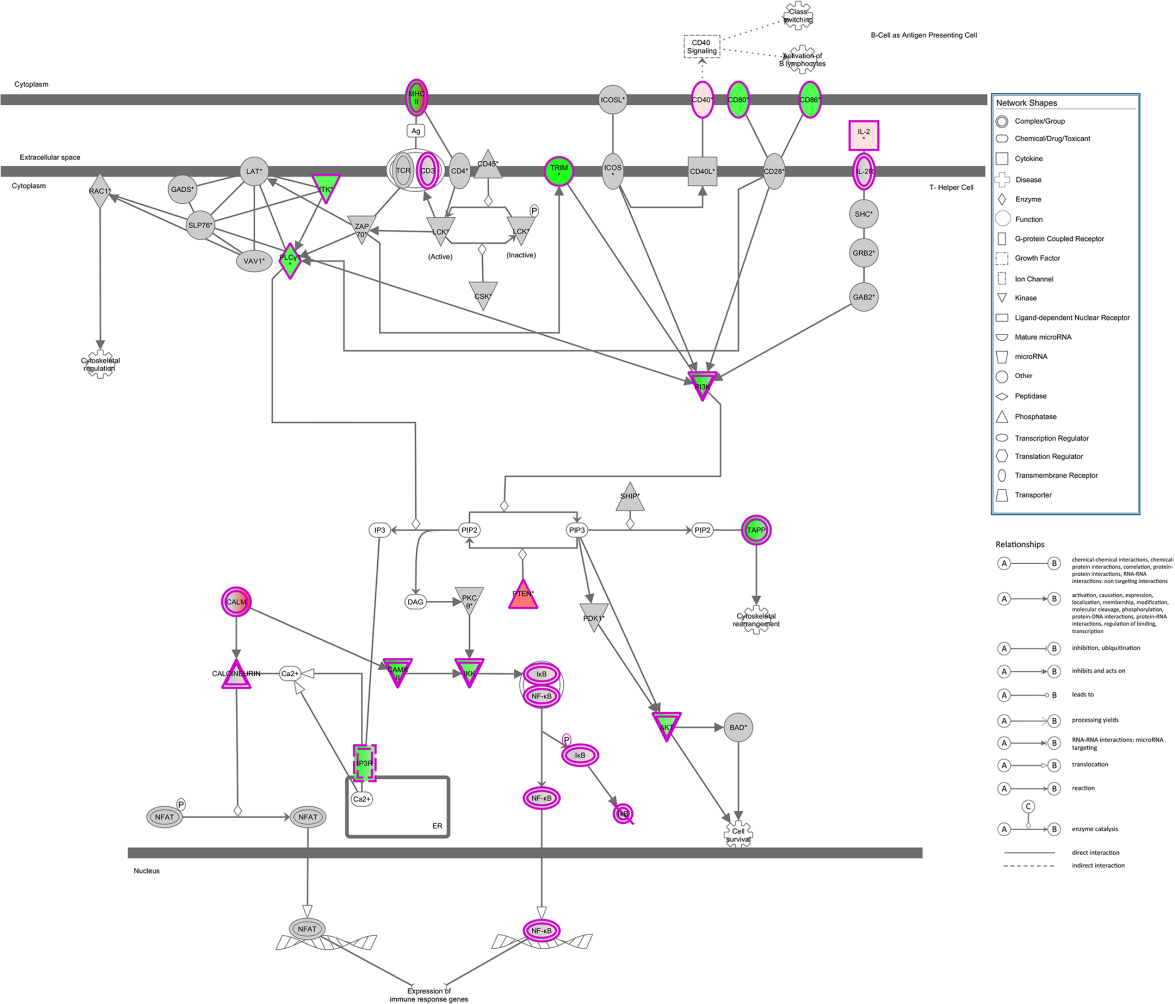
Top canonical pathway (iCOS-iCOSL Signaling in T Helper Cells) overlaid with hits from union of RIP-Seq and RNA-Seq analyses. Significant pathway nodes are shaded according to size of fold change (red >1; green <1), with white nodes indicating genes that were not detected in the samples and gray indicating genes that were detected, but not significant. Nodes with multicolor gradients denote nodes with significant genes with fold changes in different directions (see Table 2 for more details). Fold change is defined as KO/CTL (RNA-Seq) or IP/CTL (RIP-Seq). Colored double borders indicate that the molecule is a complex.

**Fig 3:**
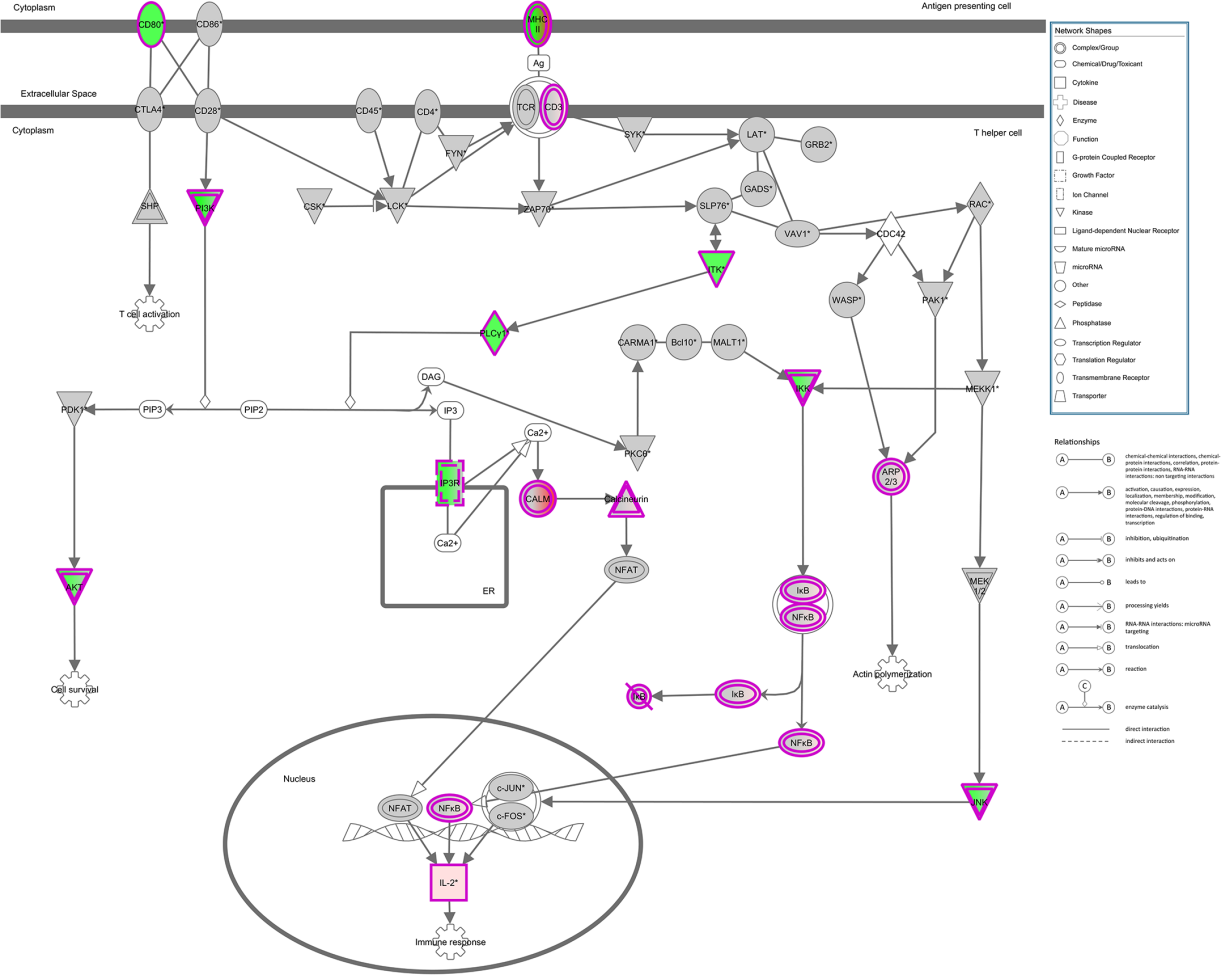
Canonical pathway for CD28 Signaling in T Helper Cells overlaid with hits from union of RIP-Seq and RNA-Seq analyses. Significant pathway nodes are shaded according to size of fold change (red >1; green <1), with white nodes indicating genes that were not detected in the samples and gray indicating genes that were detected, but not significant. Nodes with multicolor gradients denote nodes with significant genes with fold changes in different directions (see Table 3 for more details). Fold change is defined as KO/CTL (RNA-Seq) or IP/CTL (RIP-Seq). Colored double borders indicate that the molecule is a complex.

Based on the union of the RNA-Seq and RIP-Seq lists, we used IPA software to identify the top 50 enriched canonical pathways (Table 4). We reported the significance and indicated the magnitude of the enrichment as a percent of the genes in our list for a given canonical pathway divided by the size of the canonical pathway. Fig 2 gives details for the top identified canonical pathway (iCOS-iCOSL Signaling in T Helper Cells) overlaid with relevant data (Table 5). Significant pathway nodes are shaded according to the size of the fold change (red >1; green <1), with white nodes indicating undetected genes and gray indicating genes that were detected, but not significant. Nodes with multicolor gradients contain genes with significant fold changes in different directions; complete relevant data for every node is given in Table 5, Fig 3 and Table 6 present similar information for another highly significant canonical pathway, CD28 Signaling in T Helper Cells.

**Table 4:** Top 50 canonical pathways for union of RIP-Seq and RNA-Seq analyses sorted by p-value. Size indicates the number of molecules that comprise a pathway and DE indicates the number of significant genes identified by either RIP-Seq or RNA-Seq, where % represents DE/size. *p* is based on Fisher’s Exact Test and indicates pathways that are enriched for genes from RIP-Seq or RNA-Seq.

Ingenuity Canonical Pathways	*p*	DE	Size	%
iCOS-iCOSL Signaling in T Helper Cells	4.07E-07	28	126	22
Type I Diabetes Mellitus Signaling	7.94E-07	27	121	22
Mitochondrial Dysfunction	9.33E-06	34	201	17
PI3K Signaling in B Lymphocytes	4.17E-05	27	143	19
Cell Cycle: G1/S Checkpoint Regulation	8.13E-05	16	68	24
Thrombin Signaling	1.48E-04	34	211	16
CD28 Signaling in T Helper Cells	1.58E-04	24	136	18
Antioxidant Action of Vitamin C	2.34E-04	21	110	19
Production of Nitric Oxide and Reactive Oxygen Species in Macrophages	2.40E-04	32	212	15
Role of NFAT in Cardiac Hypertrophy	3.24E-04	32	209	15
T Helper Cell Differentiation	3.72E-04	16	72	22
Dendritic Cell Maturation	5.25E-04	30	211	14
Calcium-induced T Lymphocyte Apoptosis	5.89E-04	15	71	21
Huntington's Disease Signaling	6.17E-04	36	252	14
Role of BRCA1 in DNA Damage Response	6.76E-04	14	71	20
iNOS Signaling	6.76E-04	12	53	23
Small Cell Lung Cancer Signaling	7.08E-04	16	94	17
Glucocorticoid Receptor Signaling	8.51E-04	40	299	13
Death Receptor Signaling	1.55E-03	13	68	19
Cardiac Hypertrophy Signaling	1.62E-03	35	248	14
p70S6K Signaling	1.74E-03	22	132	17
Amyloid Processing	1.78E-03	12	61	20
RANK Signaling in Osteoclasts	1.86E-03	17	97	18
Apoptosis Signaling	1.86E-03	17	100	17
PKCθ Signaling in T Lymphocytes	2.14E-03	21	144	15
Role of PKR in Interferon Induction and Antiviral Response	2.24E-03	10	49	20
NRF2-mediated Oxidative Stress Response	2.75E-03	28	194	14
Integrin Signaling	2.75E-03	30	208	14
Glioma Signaling	3.09E-03	18	113	16
Thioredoxin Pathway	3.31E-03	4	8	50
Activation of IRF by Cytosolic Pattern Recognition Receptors	3.39E-03	13	74	18
Chronic Myeloid Leukemia Signaling	3.80E-03	17	106	16
Role of NFAT in Regulation of the Immune Response	4.27E-03	27	200	14
Cyclins and Cell Cycle Regulation	4.47E-03	15	96	16
Synaptic Long Term Potentiation	4.68E-03	21	130	16
LPS-stimulated MAPK Signaling	4.79E-03	14	83	17
4-1BB Signaling in T Lymphocytes	4.90E-03	8	36	22
Regulation of IL-2 Expression in Activated and Anergic T Lymphocytes	5.01E-03	15	89	17
IL-17A Signaling in Airway Cells	5.13E-03	13	76	17
NGF Signaling	5.25E-03	19	122	16
RAR Activation	5.37E-03	27	191	14
Induction of Apoptosis by HIV1	5.37E-03	12	67	18
Phospholipase C Signaling	5.62E-03	34	266	13
DNA Double-Strand Break Repair by Homologous Recombination	5.75E-03	5	18	28
B Cell Receptor Signaling	5.89E-03	25	175	14
Sphingosine-1-phosphate Signaling	5.89E-03	19	123	15
Gαq Signaling	6.03E-03	24	171	14
HER-2 Signaling in Breast Cancer	6.92E-03	14	82	17
Sperm Motility	7.41E-03	21	143	15
TWEAK Signaling	7.41E-03	8	39	21

**Table 5:** Details of the hits in the top canonical pathway (iCOS-iCOSL Signaling in T Helper Cells). Official symbols of the significant genes found via RNA-Seq (RIP-Seq) that are in the pathway (Fig 2) is given along with name of its corresponding node in the pathway. Fold is given as KO/CTL (RNA-Seq) or IP/CTL (RIP-Seq), and q is the false discovery rate value based on method of Benjamini-Hochberg. Genes were identified by both RNA-Seq and RIP-Seq. Numbers in parentheses and italicized denote data obtained from RIP-Seq.

Symbol	Member of Node	Entrez Gene Name	RNA (RIP) Fold	RNA (RIP) q
AKT1	AKT	v-akt murine thymoma viral oncogene homolog 1	0.887	0.140
ATM	ATM	ataxia telangiectasia mutated	0.905	0.189
Calm1	CALM	calmodulin 1	1.132	0.153
CAMK4^†^	CALM	calcium/calmodulin-dependent protein kinase IV	0.85 (637)	0.06 (<1E-99)
Camk2b	CAMKII	calcium/calmodulin-dependent protein kinase II, beta	0.500	0.004
CD40	CD40	CD40 molecule, TNF receptor superfamily member 5	1.765	0.100
CD80	CD80\CD86	CD80 molecule	0.861	0.159
CD3D	CD3	CD3d molecule, delta (CD3-TCR complex)	1.184	0.108
CD3G	CD3	CD3g molecule, gamma (CD3-TCR complex)	1.178	0.026
HLA-DMA*	MHC II	major histocompatibility complex, class II, DM alpha	(2196)	(<1E-99)
HLA-DMB	MHC II	major histocompatibility complex, class II, DM beta	1.215	0.249
HLA-DOA	MHC II	major histocompatibility complex, class II, DO alpha	1.194	0.263
HLA-DOB	MHC II	major histocompatibility complex, class II, DO beta	0.622	<1E-3
IKBKE	IKK	inhibitor of kappa light polypeptide gene enhancer in B-cells, kinase epsilon	0.868	0.244
IKBKG	IKK	inhibitor of kappa light polypeptide gene enhancer in B-cells, kinase gamma	0.935	0.282
IL2	IL-2	interleukin 2	1.933	0.103
IL2RG	IL-2R	interleukin 2 receptor, gamma	1.167	0.036
ITK	ITK	IL2-inducible T-cell kinase	0.912	0.244
ITPR1	IP3R	inositol 1,4,5-trisphosphate receptor, type 1	0.806	0.221
ITPR3	IP3R	inositol 1,4,5-trisphosphate receptor, type 3	0.881	0.258
NFKB2	NFκB	nuclear factor of kappa light polypeptide gene enhancer in B-cells 2 (p49/p100)	1.096	0.285
NFKBIE	IκB	nuclear factor of kappa light polypeptide gene enhancer in B-cells inhibitor, epsilon	1.089	0.111
PIK3C2A	PI3K	phosphatidylinositol-4-phosphate 3-kinase, catalytic subunit type 2 alpha	0.627	0.017
PLCG1	PLCγ1	phospholipase C, gamma 1	0.891	0.220
PLEKHA4	TAPP	pleckstrin homology domain containing, family A (phosphoinositide binding specific) member 4	0.706	0.214
PPP3CA	Calcineurin	protein phosphatase 3, catalytic subunit, alpha isozyme	1.264	0.049
PTEN*	PTEN	phosphatase and tensin homolog	(228)	(<1E-99)
TRAT1	TRIM	T cell receptor associated transmembrane adaptor 1	0.589	0.038

*found only by RIP-Seq

^†^found by both RNA-Seq and RIP-Seq

**Table 6:** Details of the hits of canonical pathway CD28 Signaling in T Helper Cells. Official symbols of the significant genes found via RNA-Seq (RIP-Seq) that are in the pathway (Fig 3) is given along with the name of its corresponding node in the pathway. Fold is given as KO/CTL (RNA-Seq) or IP/CTL (RIP-Seq), and q is the false discovery rate value based on method of Benjamini-Hochberg. Genes were identified by both RNA-Seq and RIP-Seq. Numbers in parentheses and italicized denote data obtained from RIP-Seq.

Symbol	Member of Node	Entrez Gene Name	RNA (*RIP*) Fold	RNA (*RIP*) q
AKT1	AKT	v-akt murine thymoma viral oncogene homolog 1	0.887	0.140
ARPC4	ARP2/3	actin related protein 2/3 complex, subunit 4, 20kDa	1.108	0.088
ATM	ATM	ataxia telangiectasia mutated	0.905	0.189
Calm1	CALM	calmodulin 1	1.132	0.153
CAMK4^†^	CALM	calcium/calmodulin-dependent protein kinase IV	0.85 (*637*)	0.06 (<*1E-99*)
CD80	CD80/CD86	CD80 molecule	0.861	0.159
CD3D	CD3	CD3d molecule, delta (CD3-TCR complex)	1.184	0.108
CD3G	CD3	CD3g molecule, gamma (CD3-TCR complex)	1.178	0.026
HLA-DMA*	MHC II	major histocompatibility complex, class II, DM alpha	(*2196*)	(*<1E-99*)
HLA-DMB	MHC II	major histocompatibility complex, class II, DM beta	1.215	0.249
HLA-DOA	MHC II	major histocompatibility complex, class II, DO alpha	1.194	0.263
HLA-DOB	MHC II	major histocompatibility complex, class II, DO beta	0.622	<1E-3
IKBKE	IKK	inhibitor of kappa light polypeptide gene enhancer in B-cells, kinase epsilon	0.868	0.244
IKBKG	IKK	inhibitor of kappa light polypeptide gene enhancer in B-cells, kinase gamma	0.935	0.282
IL2	IL-2	interleukin 2	1.933	0.103
ITK	ITK	IL2-inducible T-cell kinase	0.912	0.244
ITPR1	IP3R	inositol 1,4,5-trisphosphate receptor, type 1	0.806	0.221
ITPR3	IP3R	inositol 1,4,5-trisphosphate receptor, type 3	0.881	0.258
MAPK9	JNK	mitogen-activated protein kinase 9	0.864	0.010
NFKB2	NFκB	nuclear factor of kappa light polypeptide gene enhancer in B-cells 2 (p49/p100)	1.096	0.285
NFKBIE	IκB	nuclear factor of kappa light polypeptide gene enhancer in B-cells inhibitor, epsilon	1.089	0.111
PIK3C2A	PI3K	phosphatidylinositol-4-phosphate 3-kinase, catalytic subunit type 2 alpha	0.627	0.017
PLCG1	PLCγ1	phospholipase C, gamma 1	0.891	0.220
PPP3CA	Calcineurin	protein phosphatase 3, catalytic subunit, alpha isozyme	1.264	0.049

Multiple pathways involved in CD4^+^ T cell activation and Th2 differentiation are displayed in Tables 3 to 6 and Figs 2 and 3. Two major CD28 family co-stimulatory pathways, iCOS-iCOSL (Inducible Co-stimulator)/AILIM (Activation-inducible lymphocyte immunomediatory molecule) and CD28, in T helper cells are the most effected by HuR ablation. iCOS and CD28 are both co-stimulatory molecules which are highly up-regulated upon the engagement of T cell receptor (TCR). iCOS is induced upon T cell activation but not in naïve CD4^+^ T cells while CD28 is constitutively expressed even before TCR engagement [24, 25]. iCOS has been shown to be essential for Th2 differentiation and for follicular helper T cell (Tfh) development while CD28 signal is required for IL-2 production in activated CD4^+^ T cells [26–28]. This data emphasize the possibility that HuR may play a role in regulating CD28 co-stimulatory receptor family signaling pathways which control T cell function and differentiation.

In order to verify several potential HuR target transcripts that have been illustrated previously to be involved in the iCOS-iCOSL pathway, we have quantitated CAMK2, CAMK4 and IL-2 levels in our system. CAMK2 and CAMK4 have been shown previously to inhibit IL-2 transcription [29]. Interestingly, our data show potential direct and indirect HuR regulation of CAMK2 and CAMK4 on IL-2 expression. Down regulation of CAMK2 and CAMK4 observed in Th2 differentiated HuR KO CD4^+^ T cells may result in up-regulation of IL-2 expression. Although the mRNA levels of CAMK2 were significantly lower in the HuR KO Th2 polarized CD4^+^ T cells as compared to the control, the protein levels were not significantly different (S1A–S1D Fig). This may be due to the inability of Western blotting to detect subtle changes in protein expression. Also, there are 4 isoforms of CAMK2 (α, β, δ and γ). Two isoforms of CAMK2 (β and β’e splice variants) have been shown to suppress IL-2 expression [29]. The antibody used in this experiment detects all CAMK2 isoforms, making it difficult to detect subtle alterations in individual isoform expression. Moreover, only expression of the CAMK2b isoform was altered in the absence of HuR and was a HuR target. These results emphasize the fact that HuR regulation of target mRNA transcripts is complex. Additionally, HuR regulation at the translation levels has to be considered in interpretation of mRNA results. Therefore, further experiments are needed to confirm the posttranscriptional regulation of these potential HuR regulated genes.

## Discussion

It has long been appreciated that there is a poor correlation between steady state mRNA levels and protein products [30, 31]. This observation strongly implied the importance of posttranscriptional gene regulation by RBPs and miRNAs. Keene and colleagues proposed the posttranscriptional operon hypothesis and developed techniques to identify *in vivo* mRNA targets of different RBPs [32–35]. These techniques have been used by many labs to delineate the universe of mRNA transcripts which co-exist in ribonucleoparticles (RNPs) with ribosomes, RBPs and miRNAs [1, 36–38]. The further development of PAR-CLIP by Tuschl and his group allowed for fine mapping of direct residues which RBPs used to interact with their targets [3, 39, 40]. Taken together, these approaches have greatly aided the field in better delineation of posttranscriptionally regulated gene networks. Furthermore, these methods identified mRNAs which may have been previously over-looked using traditional profiling methods since their steady-state mRNA levels did not appreciably change.

As suggested by Phil Sharp and colleagues, RBPs and miRNA interact to affect cellular gene expression programs to help cells survive environmental stresses [41]. However, it has become increasingly clear that the cell employs these same mechanisms to posttranscriptionally control gene expression during differentiation and development in many tissues. More recent works have identified the existence non-coding RNAs (such as lincRNAs and others) and have strongly suggested important roles for these evolutionally conserved genes in biology.

Many early analyses of RNP structure using RIP techniques were performed with HuR and family members since they are stabilizer RBPs, making it much easier to recover low copy number mRNA targets [1, 5]. However, these approaches have been expanded to include other RNA binding proteins [42, 43]. Data from different groups indicate that HuR interacts with miRNAs in a cell specific context to either positively or negatively regulate target gene expression [44, 45]. Many of the previous works were limiting in that transformed cancer cell lines were used. Few studies were performed in primary cells or using tissues from animal models. However, to identify physiologically relevant immune regulation, it was imperative to expand utility of these techniques to *in vivo* animal models using primary cells. Another advantage of HuR genetic ablation approaches is that one can generate very low levels of HuR to achieve essentially a null allele in tissues, which can be difficult with conventional siRNA approaches.

It had been previously demonstrated that HuR plays crucial roles in cancer and immune cells, especially during T cell activation and development. We used a novel conditional HuR KO which ablates HuR following T cell activation since earlier ablation may interfere with T cell development and potentially alter gene expression profiles [7]. We focused upon CD4^+^ T cell differentiation since our earlier work as well as from other groups, has demonstrated the significance of HuR in T cell cytokine expression [7, 8, 46, 47].

We analyzed data using Ingenuity Pathway Analysis (IPA) to identify top canonical pathways in which multiple genes were affected to probe HuR function in Th2 differentiated CD4^+^ T cells. To our knowledge, this is first report studying direct and indirect HuR immune targets from primary CD4^+^ T cells using high coverage next generation sequencing. Our results identify many direct and indirect HuR gene targets which may play important roles in T cell activation and Th2 differentiation. We have verified not only various HuR targets common among other cell types but have also discovered novel HuR target transcripts specific for Th2 cell differentiation. Our data show that HuR may be involved in multiple T cell activation pathways especially in CD28 family co-stimulatory molecule signaling pathways. However, we note care in interpreting the data, since RNP formation has both kinetic and spatial components so that timing of HuR ablation may alter target milieu. It would be prudent to confirm any putative HuR mRNA targets identified by RIP-Seq and RNA-Seq at the level of translation. Indeed, a recent publication has used an RNA-Seq heavy polysome screen to identify translationally active HuR targets [48].HuR may function differently in distinct cell types since the mRNA and miRNAs expression milieu varies. In future studies, it will be important to vary timing of HuR ablation and determine the effects, if any, upon direct and indirect mRNA targets.

In summary, our data combined with studies from other labs can help delineate posttranscriptional networks by which RBPs, miRNAs and lincRNAs can coordinately affect cellular gene expression at different points. Such information can directly aid in our understanding of many pro-inflammatory disease processes such as asthma and autoimmunity.

## Supporting Information

S1 Fig a-c: Data representative of three HuR target transcripts altered in the absence of HuR. IL-2 (a), CAMK2 (b) and CAMK4 (c) mRNA levels in Th2 polarized cells on day 5 post-activation. mRNA from Th2 polarized cells from OX40-*cre* HuR^fl/fl^ (KO) or control mice were isolated and analyzed for IL-2, CAMK2 and CAMK4 levels by RT-qPCR. The data were normalized to non-HuR target control GAPDH mRNA. n = 3, *p<0.05, **p<0.001. (D): Western blot analysis shows levels of HuR, CAMK2 and β-actin (loading control) proteins in unactivated KO (lane1), unactivated control (lane2), Th2 polarized KO (lane3) and Th2 polarized control cells (lane4).
(TIF)

S2 Fig a: CAMK4 and IL-2 are HuR target transcripts in Th2 polarized cells. HuR RNA-Immunoprecipitation (HuR-RIP) assay for detection of CAMK2, CAMK4 and IL-2 mRNA enrichment in Th2 polarized extracts immunoprecipitated with HuR or IgG1 isotype antibodies followed by RT-qPCR. n = 2, **p<0.01.
(TIF)

S1 Table Full list of genes which are indirect HuR targets in CD4^+^ Th2 differentiation identified by RNA-Seq. (XLSX)

S2 Table Full list of genes which are direct *in vivo* HuR targets identified by RIP-Seq in CD4^+^ Th2 differentiation. (XLSX)
