# Supplementary figures and images for: Transcriptomic-Wide Discovery of Direct and Indirect HuR RNA Targets in Activated CD4+ T Cells
Source: PLoS One. 2015 Jul 10;10(7):e0129321. doi: 10.1371/journal.pone.0129321 (PMC4498740; doi:10.1371/journal.pone.0129321)

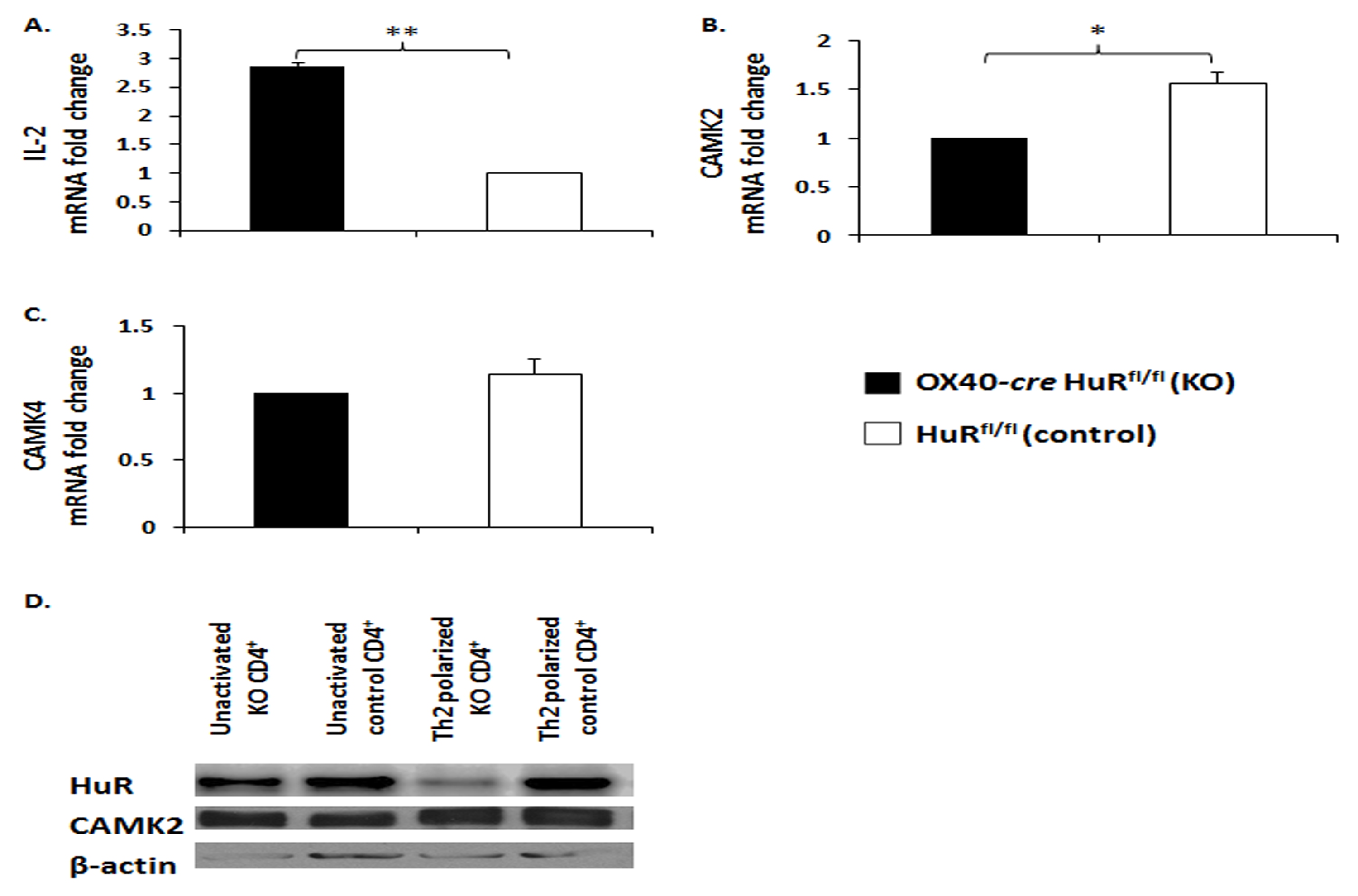

Supplement: S1 Fig — IL-2 (a), CAMK2 (b) and CAMK4 (c) mRNA levels in Th2 polarized cells on day 5 post-activation. mRNA from Th2 polarized cells from OX40-cre HuRfl/fl (KO) or control mice were isolated and analyzed for IL-2, CAMK2 and CAMK4 levels by RT-qPCR. The data were normalized to non-HuR target control GAPDH mRNA. n = 3, *p<0.05, **p<0.001. (D): Western blot analysis shows levels of HuR, CAMK2 and β-actin (loading control) proteins in unactivated KO (lane1), unactivated control (lane2), Th2 polarized KO (lane3) and Th2 polarized control cells (lane4). (TIF) [file pone.0129321.s001.tif]

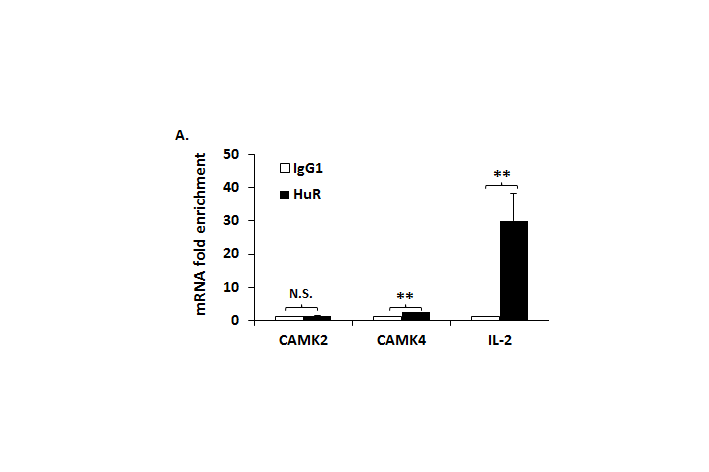

Supplement: S2 Fig — HuR RNA-Immunoprecipitation (HuR-RIP) assay for detection of CAMK2, CAMK4 and IL-2 mRNA enrichment in Th2 polarized extracts immunoprecipitated with HuR or IgG1 isotype antibodies followed by RT-qPCR. n = 2, **p<0.01. (TIF) [file pone.0129321.s002.tif]
